# Reference LINE-1 insertion polymorphisms correlate with Parkinson’s disease progression and differential transcript expression in the PPMI cohort

**DOI:** 10.1038/s41598-023-41052-1

**Published:** 2023-08-24

**Authors:** Alexander Fröhlich, Abigail L. Pfaff, Vivien J. Bubb, John P. Quinn, Sulev Koks

**Affiliations:** 1 Department of Pharmacology and Therapeutics, Institute of Systems, Molecular and Integrative Biology, University of Liverpool, Liverpool, UK; 2 Perron Institute for Neurological and Translational Science, Perth, WA Australia; 3 Centre for Molecular Medicine and Innovative Therapeutics, Murdoch University, Perth, WA Australia

**Keywords:** Neuroscience, Parkinson's disease, Gene expression, Gene regulation, Genotype

## Abstract

Long interspersed nuclear element-1 (LINE-1/L1) retrotransposons make up 17% of the human genome. They represent one class of transposable elements with the capacity to both mobilize autonomously and in *trans* via the mobilization of other elements, primarily *Alu* and SVA elements. Reference LINE-1 elements are, by definition, found in the reference genome, however, due to the polymorphic nature of these elements, variation for presence or absence is present within the population. We used a combination of clinical and transcriptomic data from the Parkinson’s Progression Markers Initiative (PPMI) and applied matrix expression quantitative trait loci analysis and linear mixed-effects models involving 114 clinical, biochemical and imaging data from the PPMI cohort to elucidate the role of reference LINE-1 insertion polymorphism on both gene expression genome-wide and progression of Parkinson’s disease (PD). We demonstrate that most LINE-1 insertion polymorphisms are capable of regulating gene expression, preferentially in *trans*, including previously identified PD risk loci. In addition, we show that 70 LINE-1 elements were associated with longitudinal changes of at least one PD progression marker, including ipsilateral count density ratio and UPDRS scores which are indicators of degeneration and severity. In conclusion, this study highlights the effect of the polymorphic nature of LINE-1 retrotransposons on gene regulation and progression of PD which underlines the importance of analyzing transposable elements within complex diseases.

## Introduction

**Figure 1:**
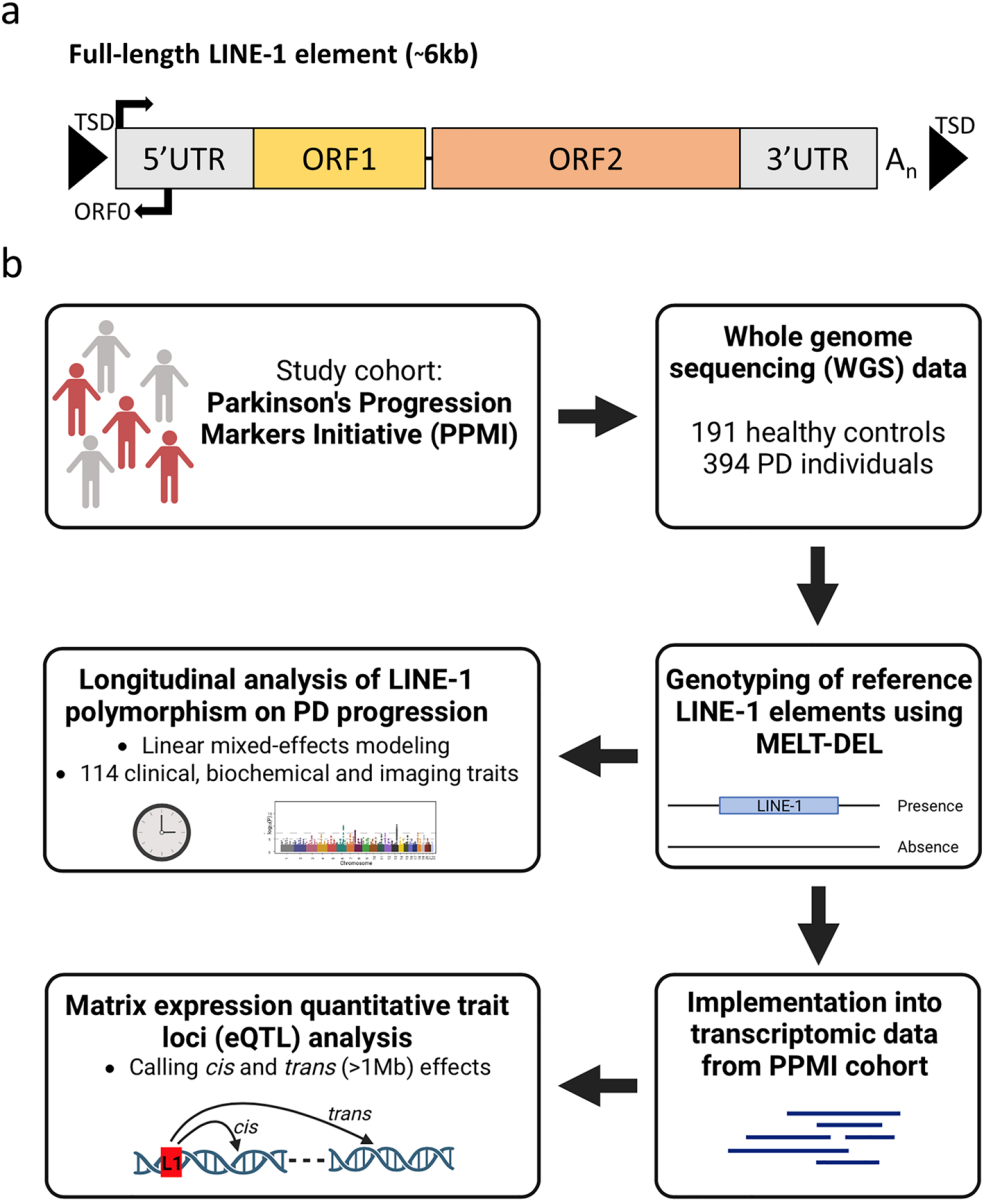
Structure of LINE-1 element and overview of study. (**a**) A full-length LINE-1 element is about 6kb in length and consists of a 5’ and 3’ untranslated region (UTR), three open reading frames (ORF0, ORF1, ORF2) and a poly(A) tail. The element is flanked by target site duplications (TSDs). The 5’UTR involves a sense and antisense promoter. (**b**) In this study, a combination of whole genome sequencing and transcriptomic datasets from the Parkinson’s Progression Markers Initiative (PPMI) was used. Aim of the study was to associate reference LINE-1 polymorphism with Parkinson’s disease progression and to assess its role as expression quantitative trait loci (eQTL).

Long interspersed nuclear element 1 (LINE-1/L1) retrotransposons represent transposable elements which are capable of autonomous propagation throughout the genome via a “copy-&-paste” mechanism, called target-primed reverse transcription (TPRT)^1,2^. A full-length LINE-1 element is about 6kb in length and consists of a 5′ untranslated region (UTR), three open reading frames (ORF0, ORF1, and ORF2), a 3′UTR, and a poly A-tail (Fig. 1a)^3^. Through the process of TPRT, new LINE-1 insertions are flanked 5’ and 3’ by duplicated sequences called target site duplications (TSD) which are regarded as hallmarks of LINE-1 mediated retrotransposition^3^. While LINE-1 elements contribute to 17% of the human genome (more than 1 million copies present), only a small subset of 146 elements are full-length; in other words, most LINE-1 elements are 5’ truncated and therefore unable to mobilize^4,5^. LINE-1 insertion events can contribute to genome variability within the population and can also lead to disease^6–8^. To date, more than 124 LINE-1-mediated retrotransposition events can be attributed to a variety of diseases, caused by dysfunctional splicing, exon skipping and double strand DNA breaks among others^6–8^. Previous studies have established a connection between the increase in LINE-1 activity and a heightened risk of developing Parkinson's disease (PD). Nielsen et al. conducted a study in 2012 that associated smoking with the loss of LINE-1 DNA methylation, which can result in the de-repression of LINE-1 and potentially increase the risk of PD^9^. Another study indicated that mitochondrial stress may also trigger LINE-1 activation by causing a loss of LINE-1 methylation^10^. Here, the induction of reactive oxygen species production resulted in reduced levels of LINE-1 methylation, leading to the activation of LINE-1 expression via de-repression^10^.

In 2019, Nalls et al. conducted the largest genome-wide association study (GWAS) for PD to date^11^. The authors identified 90 independent risk signals; in addition, LD score regression (LDSC) and polygenic risk scores (PRS) estimated that 26–36% of PD's genetic predisposition could be attributed to the identified variants, highlighting the significant genetic contribution to PD development^11^. However, a substantial proportion of the heritability remains unexplained. Therefore, other genomic elements, such as repetitive DNA in form of LINE-1 retrotransposons, which are frequently excluded in bioinformatic studies, may account for the missing heritability not detected by GWAS. A more detailed and targeted analysis is required to understand the role of transposable elements within PD and other neurodegenerative diseases. Billingsley et al. performed a genome-wide structural variant analysis by testing over 3,154 structural variant loci in PD which increased the understanding of genetic variations at PD risk loci^12^.

Prior research has demonstrated the potential for analyzing transposable elements (TEs) in the context of complex diseases, with a focus on studying their presence or absence in the genome, termed retrotransposon insertion polymorphism (RIP). For example, an SVA insertion in the *TAF1* gene's intron was found to cause X-linked Dystonia Parkinsonism (XDP)^13–15^. Our recent studies also suggest that polymorphic non-reference transposable elements and reference SVAs are involved in PD progression^16,17^. In the present study, we focused on reference LINE-1 insertion polymorphisms, which means that these elements are generally found in the human reference genome, however, these elements can be polymorphic for their presence or absence in the population. For analysis, we used the Parkinson's Progression Markers Initiative (PPMI) cohort which allows the impact of LINE-1 elements in PD traits due to its availability of clinical, imaging, transcriptomic and whole-genome sequencing (WGS) data (Fig. 1a). We combined available datasets and reference LINE-1 genotypes in order to elucidate the role of LINE-1 insertion polymorphism on both global gene expression and progression of PD (Fig. 1b). We demonstrate that LINE-1 insertion polymorphisms are capable of significantly regulating gene expression, preferentially in *trans*, including previously identified PD risk loci. These involve transcripts encoded by inositol polyphosphate-5-phosphatase F (*INPP5F*), potassium voltage-gated channel modifier subfamily S member 3 (*KCNS3*) and golgi brefeldin A resistant guanine nucleotide exchange factor 1 (*GBF1*). In addition, we show that 70 LINE-1 elements were associated with longitudinal changes of at least one PD progression marker, including ipsilateral count density ratio and UPDRS scores. Taken together, this study highlights the effect of the polymorphic nature of reference LINE-1 retrotransposons on gene regulation and progression of PD which underlines the importance of analyzing transposable elements within complex diseases and their missing heritability.

## Results

### Reference LINE-1 insertion polymorphisms correlate with PD progression

In order to analyse the impact of reference LINE-1 retrotransposons on PD progression, we correlated genotypes from 116 LINE-1 retrotransposons with longitudinal data from the PPMI cohort. This involves 114 clinical, biochemical and imaging traits from up to five visits (Baseline/BL, V04, V06, V08, V10, V12). Here we demonstrate that in total 72 clinical progression markers (clinical scores or traits) were affected by LINE-1 insertion polymorphism; in other words, presence or absence of reference LINE-1 retrotransposons significantly correlated with this number of clinical progression markers (FDR *P* < 0.05).

**Figure 2:**
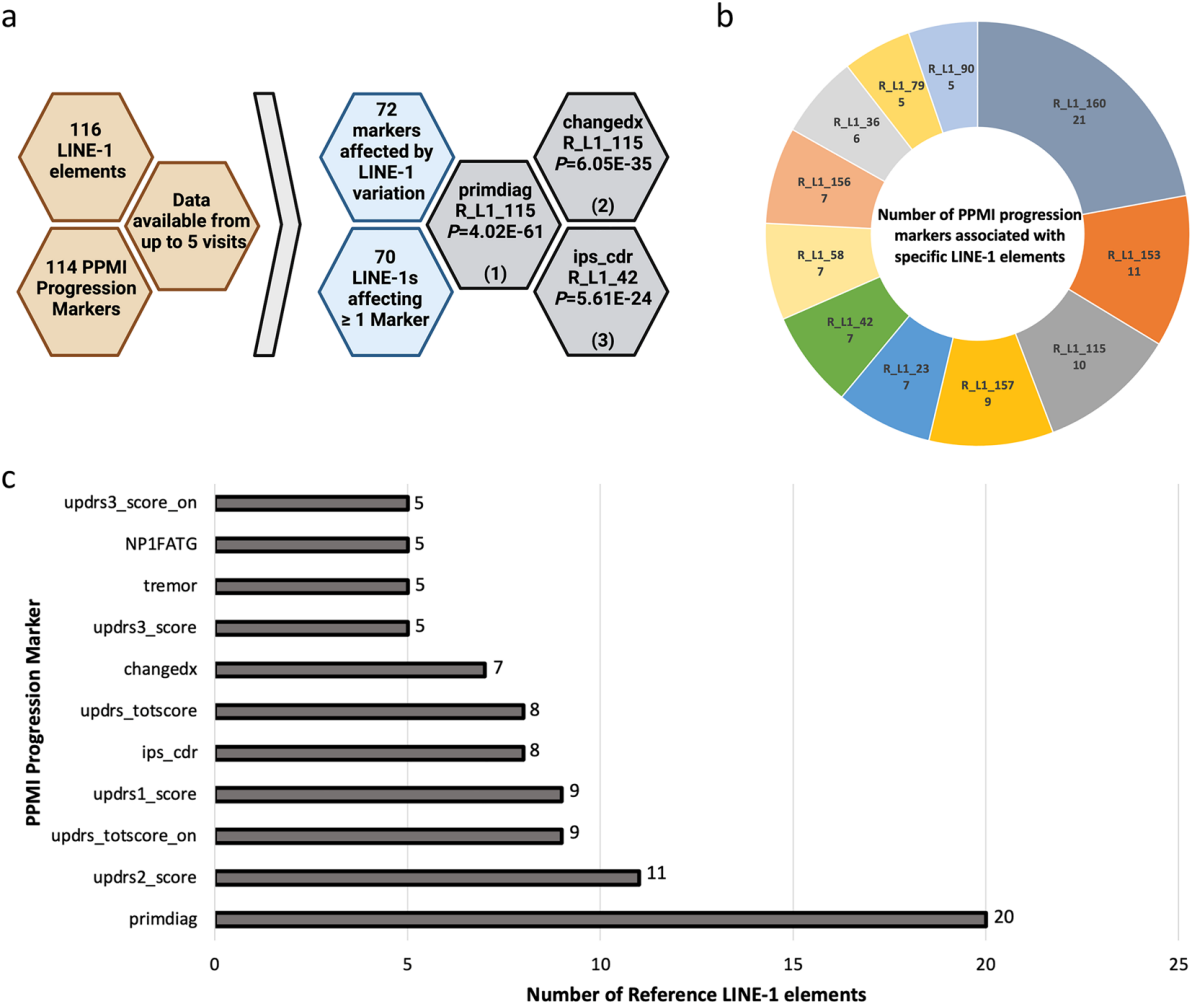
Reference LINE-1 elements are associated with PPMI progression markers. (**a**) Longitudinal and clinical data from the PPMI cohort was used for association analysis. 70/116 LINE-1 elements were associated with at least one progression marker. Top three hits were associated with primary diagnosis (primdiag), change in primary diagnosis (changedx) and ipsilateral count density ratio (ips_cdr). FDR corrected *P* values are shown. (**b**) Pie chart representing the total number of PPMI progression markers associated with a single LINE-1 element. R_L1_160 correlated with the highest number of markers (21). (**c**) Bar chart displaying the number of LINE-1 elements associated with a single PPMI progression marker. Only progression markers with > 5 hits are shown. Updrs3_score_on, MDS-UPDRS Part III Score (ON); NP1FATG, MDS_UPDRS Part I Fatigue; tremor, Tremor Score (OFF); updrs3_score, MDS-UPDRS Part III Score (OFF); changedx, Change in Primary Diagnosis from Baseline to current visit; updrs_totscore, MDS-UPDRS Total Score (OFF); ips_cdr, Ipsilateral count density ratio; updrs1_score, MDS-UPDRS Part I Score; updrs_totscore_on, MDS_UPDRS Total Score (ON); updrs2_score (MDS-UPDRS Part II Score); primdiag, Primary Diagnosis.

Interestingly, 70 LINE-1 elements correlated with changes of at least one progression marker indicating the plethora of PD progression changes associated with LINE-1 elements (Fig. 2a). The top three significant changes were found in primary diagnosis (R_L1_115, *P* = 4.02E-61), changes in primary diagnosis (R_L1_115, *P* = 6.05E-35) and ipsilateral count density ratio (R_L1_42, *P* = 5.61E-24) (Fig. 2a). Eleven reference LINE-1 retrotransposons showed an effect on at least five progression markers, with R_L1_160 displaying the highest association with 21 markers followed by R_L1_153 with 11 markers (Fig. 2b). A full-list of LINE-1 elements significantly associating with PPMI progression markers is shown in Supplementary Data 1. In addition, eleven progression traits were affected by at least five LINE-1 elements (Fig. 2c). The highest number of LINE-1 retrotransposons were associated with primary diagnosis (20 elements). Other commonly affected markers represented MDS-UPDRS Part III Score (ON) (5 hits), MDS-UPDRS Part I Fatigue (5 hits), Tremor Score (OFF) (5 hits), MDS-UPDRS Part III Score (OFF) (5 hits), change in primary diagnosis (7 hits), MDS-UPDRS Total Score (OFF) (8 hits), ipsilateral count density ratio (8 hits), MDS-UPDRS Part I Score (9 hits), MDS_UPDRS Total Score (ON) (9 hits) and MDS-UPDRS Part II Score (11 hits) (Fig. 2c).

**Figure 3:**
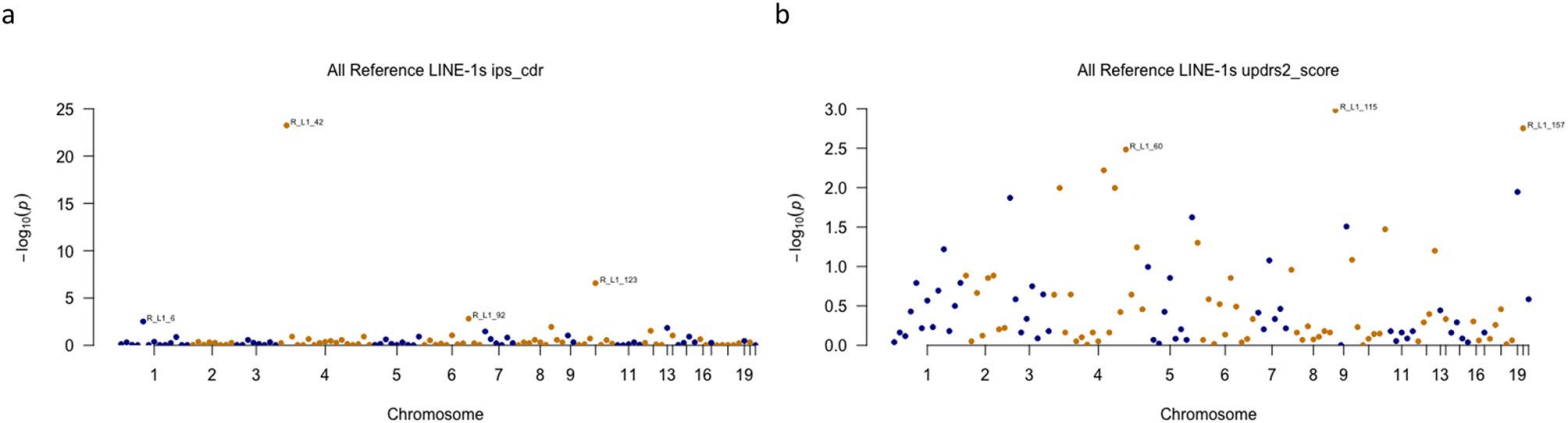
Manhattan plot for the association of all reference LINE-1 elements with PPMI progression markers. LINE-1 elements were correlated with ipsilateral count density ratio (ips_cdr) (**a**) and MDS-UPDRS Part II Score (updrs2_score) (**b**) using FDR *P* values for longitudinal changes. Chromosomal locations of these elements in the genome are indicated. Only elements with FDR *P* below 0.01 are labeled. FDR corrected *P* values are displayed on the Y-axis as –log10 values.

To expand the impact of LINE-1 elements on these progression traits, we specifically looked at the changes of ipsilateral count density ratio (IPS-CDR) and MDS-UPDRS Part II Score (UPDRS2). Figure 3 shows Manhattan plots indicating the location of LINE-1 elements and the corresponding *P* values associated with longitudinal changes of IPS-CDR (Fig. 3a) and UPDRS2 (Fig. 3b). For IPS-CDR, four elements (R_L1_6, R_L1_92, R_L1_123 and R_L1_42) showed significant association with FDR *P* < 0.01 (Fig. 3a). Similarly, UPDRS2 score was significantly (FDR *P* < 0.01) affected by the elements R_L1_60, R_L1_157 and R_L1_115 (Fig. 3b).

**Figure 4:**
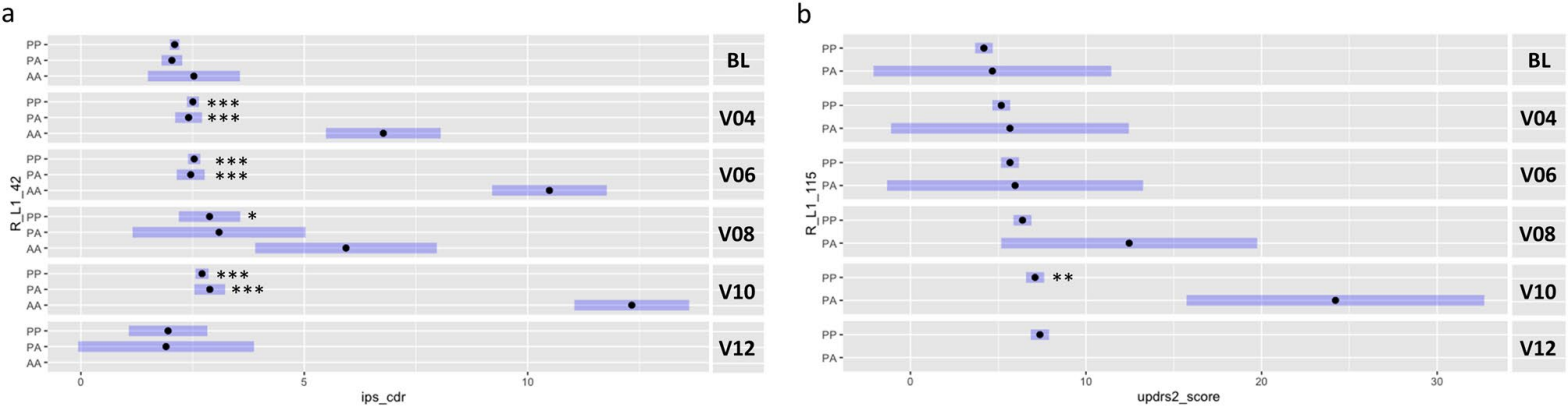
Longitudinal changes in PPMI progression markers based on LINE-1 retrotransposon insertion polymorphism. Presence/absence of specific LINE-1 elements were associated with ipsilateral count density ratio (ips_cdr, R_L1_42) (**a**) and MDS-UPDRS Part II Score (updrs2_score, R_L1_115) (**b**). Blue bars represent 95% confidence intervals. Tukey adjusted *P* values are reported compared to AA (R_L1_42) and PA (R_L1_115) genotypes. Genotypes represent the status of LINE-1 elements in the human genome in form of homozygous present (PP), heterozygous (PA) and homozygous absent (AA), respectively. Longitudinal data at baseline/BL (0 months) and up to five visits (V04/12 months, V06/24 months, V08/36 months, V10/48 months, V12/60 months)) were used for analysis. *P < 0.05, **P < 0.01, ***P < 0.001.

We next analyzed the specific effect of LINE-1 elements showing the most significant association with progression traits (R_L1_42 for IPS-CDR and R_L1_115 for UPDRS2) (Fig. 4). Figure 4A shows the longitudinal change in IPS-CDR based on presence or absence of R_L1_42. Interestingly, individuals with the absence of R_L1_42 (AA genotype) show significantly higher ipsilateral count density ratio at visits 4, 6 and 10 compared to the presence of one or two copies of this element (Fig. 4a). When looking at the change in MDS-UPDRS Part II score, only data for presence (PP genotype) or one allele missing (PA genotype) were available. However, individuals with one copy missing of R_L1_115 (PA genotype) show significantly higher UPDRS2 score at visit 10 (*P* < 0.01) which indicates that these individuals progressed faster (scores more than 10 points) and the protective role of this specific LINE-1 element regarding PD progression (Fig. 4b).

### Reference LINE-1 retrotransposons act as eQTL genome-wide

**Figure 5:**
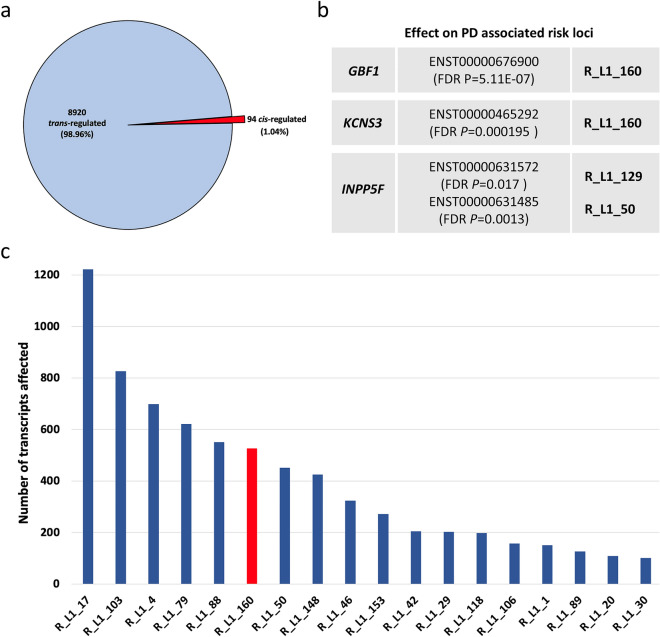
Reference LINE-1 elements act as eQTL in *cis* and *trans*. (**a**) Matrix eQTL analysis was performed in order to analyze the effect of LINE-1 elements to act as eQTL on a transcript-based level. 9014 transcripts were significantly affected (FDR *P* < 0.05) by LINE-1 insertion polymorphism, whereby the majority (98.96%) being *trans*-regulated by LINE-1 elements. (**b**) Transcripts of previously identified novel PD risk loci, including *GBF1*, *KCNS3* and *INPP5F* were significantly affected by R_L1_160, R_L1_129 and R_L1_29 polymorphism, respectively. (**c**) Bar chart showing the number of transcripts affected by specific LINE-1 elements. Only LINE-1 elements with > 100 affected transcripts are shown. R_L1_160 which was associated with the highest number of PPMI progression markers (Fig. 2) is highlighted in red.

We combined genotyping and blood-derived transcriptomic data from the PPMI cohort to analyse the capacity of reference LINE-1 elements to act as eQTL. For the analysis, we analysed 140 reference LINE-1 elements, of which 53% are located intronic and 47% intergenic (Supplementary Data 4). All LINE-1 elements analysed in this study represent members of the youngest and human- specific L1HS subfamily. We performed matrix eQTL analysis on an isoform-based level which showed that in total 9014 transcripts were significantly (FDR *P* < 0.05) affected by LINE-1 insertion polymorphisms with *P* values ranging up to 2.62E-83 (Fig. 5a). Interestingly, almost 99% of the transcripts were *trans*-regulated by LINE-1 elements (Fig. 5a) which means that the location of the corresponding affected transcript in relation to the LINE-1 element is > 1M bp. Only 94 of 9014 transcripts showed a closer location with respect to the LINE-1 element they are affected by. It is worth mentioning that only six LINE-1 elements had a *cis*-specific effect, specifically R_L1_60 (16 transcripts), R_L1_155 (9), R_L1_145 (4), R_L1_147 (3), R_L1_63 (3) and R_L1_126 (2).

When looking on the element-based level, there are 18 LINE-1 elements present which affect 100 or more transcripts genome-wide. R_L1_17 represents the element that affects the highest number of transcripts (1222) with only one transcript being *cis* regulated (Fig. 5c). Interestingly, the element R_L1_160, which previously showed to be associated with the highest number of PD progression markers (Fig. 2), affected 527 transcripts, with no transcripts regulated in *cis*. When looking specifically on the transcripts affected by R_L1_160, it can be highlighted that this element affects expression of previously identified novel PD risk loci, *GBF1* and *KCNS3* (Fig. 5b, Supplementary Data 2). Here, R_L1_160 insertion polymorphism significantly activated expression of *GBF1* transcript ENST00000676900 (FDR *P* = 5.11E-07) and *KCNS3* transcript ENST00000465292 (FDR *P* = 0.000195). Another PD risk locus represents *INPP5F*, whereby the elements R_L1_129 and R_L1_50 showed a positive effect on *INPP5F* ENST00000631572 (FDR *P* = 0.017) and ENST00000631485 (FDR *P* = 0.0013), respectively (Fig. 5b, Supplementary Data 2).

## Discussion

In this study we have analyzed the association of reference LINE-1 elements on PD progression and their ability to act as eQTL by regulating gene expression patterns based on polymorphism for presence or absence. Here, we could demonstrate that 70/116 RIP LINE-1 elements had significant influence on at least one PPMI progression marker which involve different clinical, imaging and biochemical traits. Intriguingly, the highest number of LINE-1 retrotransposon insertion polymorphisms (20 elements) were associated with primary diagnosis, in addition, more than 7 elements also correlated with change in primary diagnosis. This means that PD individuals with specific genetic variations related to LINE-1s may have a higher likelihood of initially receiving an incorrect diagnosis during early visits, which may later be corrected. This change in diagnosis could potentially highlight differences in the intermediate phenotypes between individuals with different LINE-1 genotypes. It should be emphasized that accurately diagnosing PD at the onset can be difficult due to the complex phenotype and overlapping extrapyramidal side effects^18^. The discovery that certain LINE-1s correlated with a significantly increased frequency of diagnostic challenges may reflect the genetic heterogeneity of PD and potentially propose the existence of a separate disease subtype.

In addition to that, reference LINE-1 elements also significantly correlated with UPDRS scores. These clinical scales are used to measure disease severity and include, for instance, non-motor (part I) or motor (part II) experiences of daily living. UPDRS Part II score involves measures such as an effect on speech, chewing, dressing, walking or doing activities^19^. Here we could show that presence or absence of 11 LINE-1 elements had a significant impact on the severity of this scale. To be more specifically, the element R_L1_115 had the highest impact; in other words, individuals with one copy missing of R_L1_115 (PA genotype) show significantly higher UPDRS2 score at visit 10 (*P* < 0.01) which indicates that these individuals progressed faster (scores more than 10 points compared to PP genotype) and the protective role of this specific LINE-1 element on PD progression. This difference with more than 10 points on this clinical scale may make the difference if a PD patient is able to exert the activities mentioned before or not anymore. The study presented here focused on reference LINE-1 elements, however, in our previous study we showed that non-reference (non-ref) transposable elements were also able to affect PD progression^16^. These non-ref elements are not fixed in the reference human genome and can therefore be variable for presence or absence in the genome. In total, 2886 *Alu*, 360 LINE-1, and 128 SVA elements were analysed which showed the highest number of effects on two PD progression traits, namely primary diagnosis (268 hits) and UPDRS2 score (224 hits)^16^. More specifically, when looking at non-ref LINE-1 elements, the most common traits affected were UPDRS2 score, primary diagnosis, and ipsilateral count density ratio of caudate/putamen (ips-CDR). This is consistent with our current study which showed that these traits were also commonly affected by reference LINE-1 elements supporting the effect seen on this PD progression traits.

Another PD progression marker we want to highlight here is the ipsilateral count density ratio which is based on imaging data of the brain which provides a reliable source to characterize PD progression in the form of neurodegeneration. Count density ratios (CDR) of the caudate/putamen are calculated by DaTscan single photon emission computed tomography (DaTscan SPECT) imaging that uses a radioligand to bind to the dopamine transporter (DAT) located on the presynaptic membrane of nigrostriatal neurons. The loss of DAT from the striatum is a defining characteristic of PD and is correlated with the loss of dopaminergic neurons in the substantia nigra. This loss of DAT can be used to distinguish PD from other neurodegenerative parkinsonian disorders such as essential tremor^20^. The severity of symptoms in PD is related to the reduction of striatal DAT, which changes over the course of the disease, making it an important tool for detecting dopaminergic degeneration^21,22^. The caudate/putamen ratio is a measure of the gradient of dopaminergic loss from the anterior to posterior regions, which specifically increases when the R_L1_42 is absent in the genome. It's important to note that while the LINE-1s associated with these changes may not directly cause changes to the dopaminergic loss gradient, they may be a part of the process that modifies PD disease course. One mechanism involved could be transcriptional changes of genes located in PD risk loci. Transposable elements are known to have the ability to modulate gene expression levels^23–25^; we and others have previously shown that TE insertion polymorphisms can act as eQTL and have an activating or repressive effect on transcripts in proximity (*cis*) or across chromosomes (*trans*)^26–28^. Differential gene expression triggered by the presence/absence or primary sequence variation of specific LINE-1 or other TE elements may be one mechanism by which PD course and progression can be influenced. In this study we could show that in total 9014 transcripts were significantly modified by reference LINE-1 insertion polymorphisms. Interestingly, almost 99% of these transcripts were *trans*-regulated by LINE-1 presence or absence. This is consistent with previous literature targeting the impact of TEs on gene expression which involves a study by Wang et al. which identified a greater number of *trans*-regulating TE elements using data from the 1000 genome project^27^. In our study, we used identical analysis models which increases the confidence of comparing these data with the aim to pinpoint the significant effect of transposable elements (here LINE-1 elements) on gene regulation which may also have an impact on pathways involved in neurodegeneration. It is worth mentioning that we identified transcripts encoded by previously identified PD risk loci *KCNS3*, *GBF1* and *INPP5F* ^11^ that were also affected by LINE-1 insertion polymorphism.

The results showed here benefited from the use of longitudinal data from the PPMI cohort which provides various clinical, biochemical and imaging data to monitor the course and progression of PD individuals. However, functional work in follow-up studies are necessary to validate the effects of LINE-1 insertion polymorphism. This can be achieved by analysing patient-derived cell lines harbouring the corresponding LINE-1 elements. The effects can be examined by CRISPR, followed by measurement of gene expression or cellular phenotype. Furthermore, transgenic mice can be utilised to model the impact of LINE-1 elements in form of overexpression experiments. Such an approach enables the observation of changes in brain function and behaviour, which can be tailored to the specific phenotype of Parkinson’s disease. In summary, this study described the significant impact of reference LINE-1 insertion polymorphism on 1) progression of PD by using a variety of PD progression traits, whereby a significant association with severity and degeneration of PD was observed, and 2) transcriptional regulation genome wide; thousands of transcripts were significantly *cis*- and *trans*-regulated by LINE-1 presence or absence, including transcripts encoded by PD risk genes. This study therefore highlights the plethora of transcriptomic and phenotypic changes associated with reference LINE-1 polymorphism which should be considered when analysing the role of transposable elements within neurodegenerative diseases and their missing heritability.

## Methods

### Study cohort

For this research, we obtained data from the Parkinson's Progression Markers Initiative (PPMI) cohort, which can be accessed at http://www.ppmi-info.org/data. The PPMI is a longitudinal cohort study designed to track the course of Parkinson's disease. The dataset contains 423 PD and 196 control individuals including whole transcriptome data from blood samples as well as genetic and clinical information.

### Genotyping of reference LINE-1 elements in the PPMI cohort

Reference LINE-1 elements were identified using whole-genome sequencing (WGS) data obtained from the PPMI cohort. The mobile element locator tool-deletion (MELT-DEL) (https://melt.igs.umaryland.edu/) was used to genotype reference LINE-1 elements. WGS data in bam format aligned to Hg38 were used as input for MELT-DEL. In each individual, MELT-DEL genotyped reference LINE-1 elements based on their coordinates, which were provided in a bed file. The output files were then merged to produce a final VCF. Reference LINE-1 polymorphic insertions that were not in Hardy–Weinberg equilibrium (*p* < 1 × 10E-06 in healthy controls) were removed using plink v1.07^29^. The chromosomal location and coordinates of each LINE-1 element are listed in Supplementary Data 4.

### Longitudinal analysis of reference LINE-1 elements on PD progression

The longitudinal analysis focused solely on the data of PD patients from the PPMI cohort, while control subjects' data was excluded as the aim of the study was to investigate the impact of reference LINE-1 retrotransposons on PD progression. The PPMI dataset comprises longitudinal data at baseline/BL (0 months) and up to five visits (V04/12 months, V06/24 months, V08/36 months, V10/48 months, V12/60 months) of 423 PD patients. In total, 114 clinical, imaging and biochemical traits were selected for analysis (Supplementary Data 3). The impact of the presence or absence polymorphism of LINE-1 on clinical traits was analyzed using linear mixed-effects modelling and the *LmerTest* R package. The following formula was employed for longitudinal modelling of the effect of reference LINE-1s on changes in traits between visits:

anova(lmerTest::lmer(TRAIT ~ REF-L1*months + (1|PATNO),na.action = na.omit,data = PD)).

The *P* values obtained were subjected to false discovery rate (FDR) adjustment for multiple corrections, and only FDR values below 0.05 were deemed statistically significant. The corrected FDR values were then utilized to identify significant traits for the pairwise analysis of genotype effects using the *emmeans* package in R. Pairwise comparisons *P* values were adjusted with Tukey for family-wise error rate. The use of longitudinal PPMI clinical and genomic data was approved (approval number: 2021/123) by the Human ethics committee of the Murdoch University.

### Expression quantitative trait loci (eQTL) analysis of reference LINE-1 retrotransposons

We combined determined LINE-1 genotypes with transcriptomic data obtained from the PPMI cohort to investigate the capacity of reference LINE-1 retrotransposons to act as eQTL. The Salmon tool was used to quantify transcriptomic data at the transcript-based level, and the *tximport* function from the *tximport* package was used to import the Salmon-generated quant files into R. Raw counts were extracted and then normalized using the median-of-ratios method included in the *DESeq2* package. We combined PD and healthy control individual data for the analysis. Matrix eQTL was used to determine the genetic loci regulating the expression of transcript variants. We used an additive linear model with age and sex as covariates and set the FDR threshold to 0.05. During the eQTL analysis, we called local (*cis*) and distant (*trans*) quantitative loci and set the distant locus threshold to 1M bp. The correction for multiple testing of eQTL was performed using FDR, and we only report results that remained significant after FDR correction.

### Supplementary Information

Dataset S1.

Dataset S2.

Dataset S3.

Dataset S4.

## Data Availability

The datasets used and/or analysed during the current study are publicly available from the Parkinson’s Progression Marker Initiative (www.ppmi-info.org/data).
